# *Premna puberula* P. Ethyl Acetate Extract Treats Ulcerative Colitis by Regulating the Intestinal Flora and Improving Serum Metabolism

**DOI:** 10.3390/molecules30183809

**Published:** 2025-09-19

**Authors:** Zhichao Wang, Yanmei Zhang, Yun Huang, Qiang Xiao, Yuchang Zhu, Dazhai Zhou

**Affiliations:** 1 College of Biological and Food Engineering, Hubei Minzu University, Enshi 445000, China; 202330402@hbmzu.edu.cn; 2 College of Forestry and Horticulture, Hubei Minzu University, Enshi 445000, China; 18845569341@163.com (Y.Z.); 202430445@hbmzu.edu.cn (Y.H.); 3 Hubei Key Laboratory of Biological Resources Protection and Utilization, Hubei Minzu University, Enshi 445000, China; 1992022@hbmzu.edu.cn

**Keywords:** ulcerative colitis, *Premna puberula* Pamp., UPLC-MS/MS, intestinal flora-metabolism axis, alpha-linolenic acid metabolism

## Abstract

The prevalence of Ulcerative Colitis (UC) is continuously increasing globally, demanding the urgent search for new treatment agents due to the limitations of existing therapies. *Premna puberula* Pamp. (PP), a traditional medicinal and dietary plant, has anti-inflammatory properties. Its extracts’ therapeutic benefits for UC have not been documented, though. Therefore, we aim to investigate the therapeutic effects of PPEAC on UC, providing a reference for new UC therapies. In our study, we used UPLC-MS/MS to determine the composition of *Premna puberula* Pamp’s ethyl acetate extract (PPEAC). We assessed the effectiveness of PPEAC using a UC mouse model. The results demonstrated that PPEAC significantly reduced the Disease Activity Index (DAI) scores, lowered liver and spleen weight ratios, mitigated colonic shortening and histopathological damage, and alleviated oxidative stress. This research represented the first systematic investigation into the molecular mechanism of PPEAC ameliorating UC by modulating the intestinal flora-metabolism axis. PPEAC appeared to have a therapeutic impact on UC by boosting phosphatidylcholine (PC) analog levels and the number of Firmicutes and Muribaculaceae, as well as altering alpha-linolenic acid metabolism. Our research provided a new therapeutic approach for using PP as a traditional functional plant for food and medicine, as well as a new viewpoint for the creation of UC-targeted treatments based on conventional herbs.

## 1. Introduction

Ulcerative colitis (UC), a chronic and non-specific form of inflammatory bowel disease (IBD), predominantly affects young adults between the ages of 20 and 40, with similar incidence rates observed in both males and females. It is characterized by recurrent episodes of diarrhea, muco-purulent bloody stools, and abdominal pain. The pathogenesis of UC involves a multifactorial interplay of genetic predisposition, immune system dysregulation, and alterations in gut microbiota, environmental factors, and other contributing mechanisms, all of which culminate in a disruption of intestinal mucosal immune homeostasis [1]. The main drugs used to maintain and improve the condition are Olsalazine, sulfasalazine (SF), corticosteroids and 5-aminosalicylate (5-ASA). However, its efficacy is limited, and it may cause adverse reactions such as dizziness, diarrhea, and nausea [2]. Therefore, there is an urgent need for innovative therapies that can improve efficacy and minimize adverse effects.

*Premna puberula* Pamp. (PP) is a plant of the Premna genus of the Verbenaceae family that grows mostly in southeastern China, particularly in Sichuan and Guizhou. It serves both medicinal and culinary purposes, and its leaves can be processed into the “Chinese Immortal Bean Curd,” making it a notable resource plant. As a traditional Chinese Medicine, PP’s leaves are rich in numerous metabolites, including primary metabolites such as soluble sugars, amino acids, proteins, and low-fat pectin, as well as biologically active secondary metabolites like polyphenols and flavonoids [3]. Recent studies have demonstrated that plant-derived polyphenols, flavonoids, and other bioactive molecules exhibit significant potential for alleviating UC symptoms, and their therapeutic potential can be fully exploited by extracting the active substances from plants to enhance the therapeutic efficacy further. For example, Khan et al. reported that the methanol extract of *Caralluma edulis* extracted with different solvents had the highest flavonoid content, and the extract showed good in vitro anti-inflammatory effects [4]; Garnevi Fávero et al. found that *Mimosa pudica* L. extracts had a good alleviating effect on UC in mice [5]. Liu et al. found that flavonoids in PP could reduce inflammatory factors and thus alleviate edema in mice [6]. In our preliminary study, we discovered that among the four extracts of petroleum ether, ethyl acetate, n-butanol, and water of PP, the ethyl acetate extract of PP (PPEAC) exhibited significantly superior antioxidant properties (*p* < 0.05) and demonstrated a notable bacteriostatic effect; however, its potential for treating UC has not yet been reported.

In summary, PP, a traditional dietary plant with abundant bioactive components, is expected to provide new solutions for treating UC. Afterwards, we determined the composition of PPEAC using UPLC-MS/MS. Dextran sulfate sodium salt (DSS) was employed as an inducer to establish a UC mouse model. By comparing the disease activity index (DAI), changes in organs, tissue oxidation levels, and changes in serum levels of inflammatory factors across group, we analyzed the intestinal flora-metabolite interactions in conjunction with serum metabolomics and 16S rRNA sequencing. We aim to investigate the therapeutic effect of PPEAC on UC mice and reveal its anti-UC mechanism, thereby providing a scientific basis for the development and utilization of PP in medicine.

## 2. Results

### 2.1. Identification of the Principal Components of PPEAC

PPEAC’s metabolomics were examined using LC-MS/MS without a target. The results showed that 3416 metabolites were measured, with 1679 in POS and 1737 in NEG. The total ion chromatogram (TIC) is shown in Figures S1 and S2. The identified metabolites were primarily composed of amino acids and their derivatives (833), organic acids (459), benzene and substituted derivatives (363), lipids (261), alkaloids (188), phenolic compounds (173), flavonoids (172), organic heterocyclic compounds (122), glycome (101), terpenoids (99), nucleotides and their derivatives (67), amines (64), and alcohols and other compounds (53), resulting in a total of 20 classes.

### 2.2. Therapeutic Effects of PPEAC in Mice

#### 2.2.1. Effect of PPEAC on DAI in Mice

**Figure 1:**
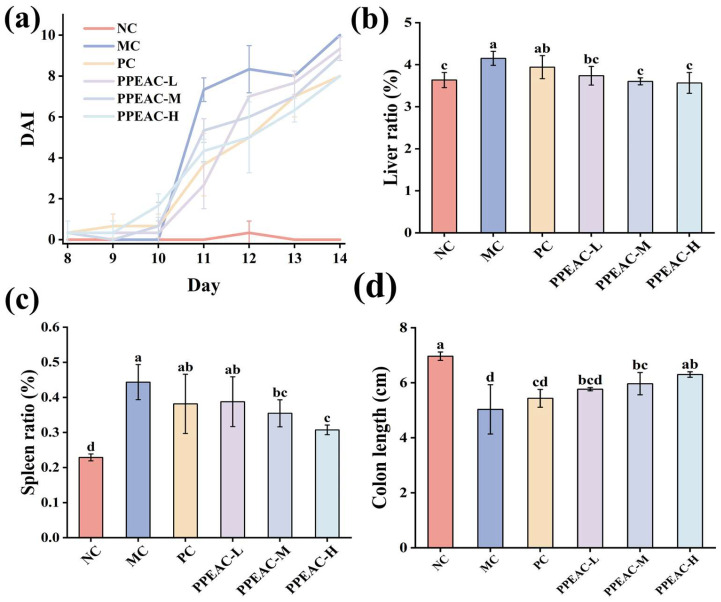
Indicators associated with colitis in mice. (**a**) DAI scores of the mouse, (**b**) Liver ratio, (**c**) Splenic ratio, (**d**) Colonic length. (Significant differences are indicated by different letters in the figure, (*p* < 0.05). Meaning of each group, NC: Normal Control—Healthy mice not treated with DSS. MC: Model Control—Mice treated with DSS to induce UC but not given any therapeutic intervention. PC: Positive Control—Mice treated with DSS and then administered 5-ASA. PPEAC-L: Low-dose PPEAC group—Mice treated with DSS and then given a low dose (125 mg/kg) of the PPEAC. PPEAC-M: Medium-dose PPEAC group—Mice treated with DSS and then given a medium dose (250 mg/kg) of the PPEAC. PPEAC-H: High-dose PPEAC group—Mice treated with DSS and then given a high dose (500 mg/kg) of the PPEAC. The same applies below).

The DAI can accurately indicate the severity of UC in mice [7]. Figure 1a depicts changes in illness severity in mice over the modeling period. The normal group’s DAI score remained constant at zero; however, following DSS-induced therapy, an inflammatory response was seen in the digestive tract of mice, and the DAI of mice in the MC, PC, PPEAC-L, PPEAC-M, and PPEAC-H groups increased. Mice in the MC group began to have loose feces on the third day of the modeling, and on the fourth day, they developed occult bleeding. As the duration of DSS treatment increased, the feces lost its specific morphology by day 6, and some mice displayed obvious clinical symptoms, such as weight loss and the presence of blood in feces, resulting in a significantly higher DAI score compared to the NC group, confirming the mice’s successful modeling. The PPEAC intervention significantly reduced diarrhea and blood in feces in all three intervention groups of mice (*p* < 0.05), and DAI scores decreased consistently, indicating that the intervention effectively mitigated the disease response induced by DSS. On the third day of treatment, PPEAC-H reduced the DAI score by 1.73% compared to PC. The PPEAC-H group saw more relief than the PC group. The PPEAC-H group showed the most significant effect.

#### 2.2.2. Effects of PPEAC on the Liver, Spleen, and Colon of Mice

Figure 1b shows that the MC group had significantly higher liver ratios compared to the NC group (increased by 0.51%, *p* < 0.05), while the PC group had lower ratios after 5-ASA treatment (it became 3.94%). PPEAC-L (3.73 ± 0.22%), PPEAC-M (3.60 ± 0.08%), and PPEAC-H (3.57 ± 0.25%) therapies significantly improved UC (*p* < 0.05) compared to the MC group (4.15%), with the PPEAC-H group having a liver ratio similar to the NC group. Figure 1c shows that mice in the MC group had significantly greater spleen index compared to the NC group (increased by 0.21%, *p* < 0.05). After PC and PPEAC interventions, the spleen index reduced in both groups, with a significant difference between the PPEAC-M and PPEAC-H groups and the MC group (*p* < 0.05), showing a reduction in spleen swelling, especially in the PPEAC-H group (0.31 ± 0.01%). Figure 1d shows that the mice in the NC group (6.97 ± 0.15 cm) had the longest colon length, whereas the animals in the MC group (5.03 ± 0.9 cm) had the shortest colon length. The colon lengths of mice in the PPEAC-M (5.97 ± 0.4 cm), PPEAC-H (6.3 ± 0.1 cm), and MC groups differed significantly (*p* < 0.05), with the PPEAC-H group having the longest and most similar colon length to the NC group.

#### 2.2.3. Effect of PPEAC on the Level of Oxidative Stress in Mouse Colon Tissues

**Figure 2:**
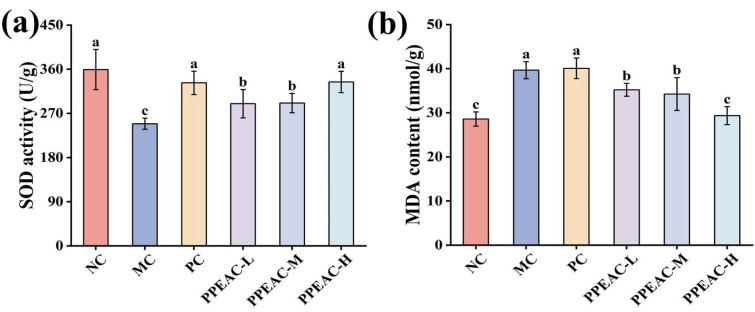
Indicators associated with colitis in mice. (**a**) SOD activity, (**b**) MDA content. (Significant differences are indicated by different letters in the figure, (*p* < 0.05)).

Superoxide Dismutase (SOD), an antioxidant enzyme that protects cells from oxidative damage by catalyzing the dismutation of superoxide radicals into oxygen and hydrogen peroxide. Figure 2a shows that SOD activity was considerably lower in the MC group compared to the NC group (decreased by 110.32 U/g, *p* < 0.05). Interventions with PPEAC-L, PPEAC-M, and PPEAC-H significantly boosted SOD activity (*p* < 0.05), with the PPEAC-H group ranking second only to the NC group (359.31 ± 41 U/g). Furthermore, PPEAC-H had the same antioxidant capacity as the PC group. Malondialdehyde (MDA), a commonly used lipid peroxidation index that serves as a biomarker for oxidative stress and cellular damage. Figure 2b shows that animals in the MC group had significantly higher levels of MDA compared to the NC group (increased by 11.07 nmol/g, *p* < 0.05), indicating that DSS exacerbated lipid peroxidation and cellular damage. PPEAC-L (35.21 ± 1.47 nmol/g), PPEAC-M (34.25 ± 3.72 nmol/g), and PPEAC-H (29.35 ± 2.04 nmol/g) therapies resulted in a substantial decrease in MDA levels (*p* < 0.05) compared to the MC group (39.66 nmol/g). There was a significant difference (*p* < 0.05) between the PPEAC-H and MC groups, and no significant change was found when compared to the NC group (28.59 ± 1.6 nmol/g).

#### 2.2.4. H&E Staining Results

**Figure 3:**
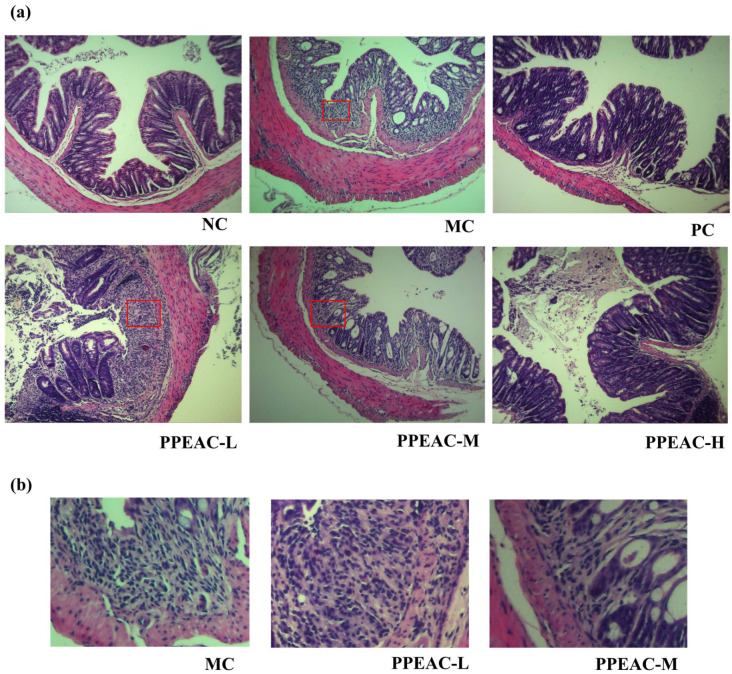
Photographs of H&E staining of mouse colon tissues (200×). (**a**) H&E staining for each group. The box highlights immune infiltration. (**b**) Local Immune Infiltration Images.

Histopathologic damage to colonic tissue was examined using H&E staining. Figure 3a,b shows that in the NC group, the epithelial structure was intact, with distinct layers of colonic tis-sue and normal gland distribution. Crypt surfaces are regular, exhibiting a pronounced U-shaped crypt morphology, with no inflammatory cell infiltration observed. The MC group exhibited partial tissue or muscularis defects, with marked mucosal hyperemia, edema, and erosion. The lamina propria and submucosa demonstrate pronounced infiltration of inflammatory cells, including neutrophils. Crypts are reduced in number and exhibit disorganized morphology. The PC group showed relatively intact mucosal architecture with markedly improved morphology approaching NC group levels. Mild inflammatory infiltration was occasionally observed, and crypts remained clearly visible. The PPEAC-L group exhibited relatively intact epithelial structure but demonstrated muscularis defects or congestion, inflammatory infiltration, and distorted crypt architecture. Although inflammatory cell aggregation persisted, its extent was reduced relative to the MC group, suggesting limited efficacy at low doses. In the PPEAC-M group, Inflammatory cell infiltration markedly decreased, though minor immune infiltration remained, indicating superior improvement over the low-dose group. The PPEAC-H group comparable to the NC and PC groups, minimal inflammatory cell infiltration approaching NC levels, and restored crypt morphology resembling the NC group, demonstrating significant anti-inflammatory effects at high doses. PPEAC improved mouse UC in a dose-dependent manner, with high-dose efficacy matching the PC group and second only to the NC group.

### 2.3. PPEAC Regulates Intestinal Flora in Mice

#### 2.3.1. Alpha Diversity and Beta Diversity of Mouse Intestinal Flora

**Figure 4:**
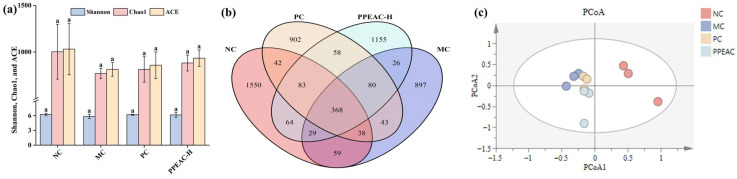
Intestinal flora of NC, MC, PC and PPEAC-H mouse. (**a**) Intestinal flora alpha diversity (Letters in the figure indicate the significance of different groups for the same metric). (**b**) Intestinal flora Venn. (**c**) Intestinal flora PCoA.

Alpha diversity represents the richness and evenness of a single sample, commonly assessed with the Shannon, ACE, and Chao1 indices [8]. A higher Shannon score and lower Simpson index indicate greater species variety. As demonstrated in Figure 4a, the MC group had lower Shannon, Chao1, and ACE indices than the NC group. Although the increase in the indices did not achieve a statistically significant difference (*p* > 0.05), the findings indicated that they had surpassed the NC group.

Beta diversity was used as an important evaluation method for assessing the similarity of the flora among the groups, and the obtained results were plotted as a Wayne diagram by cluster analysis (Figure 4b). The analysis of shared and endemic species among the groups visualized the shared and endemic species and their number in the samples. Also, it showed the similarity and overlap among the groups. The number of shared species in the NC, MC, PC, and PPEAC-H groups was 368, whereas the number of endemic species in each group was 1550, 897, 902, and 1155, respectively, indicating that the intestinal flora composition varied amongst the groups. Following PPEAC-H administration, the number of intestinal flora progressively returned to normal levels.

PCoA allowed for a better demonstration of the disparities in flora structure between the groups. Figure 4c shows that after DSS induction, there was a noticeable split between the sample groups, with the PPEAC-H group’s gut flora moving away from MC and closer to NC.

#### 2.3.2. Composition and Abundance of Mouse Intestinal Flora

**Figure 5:**
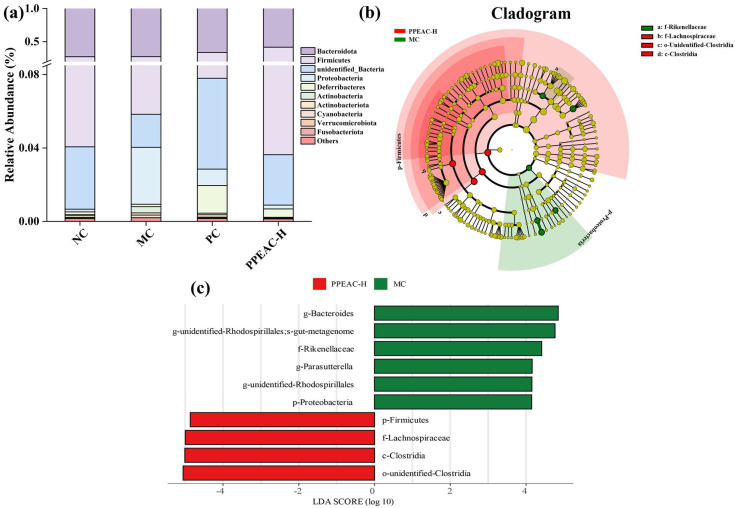
Intestinal flora of NC, MC, PC and PPEAC-H mouse. (**a**) Histogram of abundance at the phylum level of the mouse intestinal flora. (**b**) Evolutionary branching diagram. Circles radiating from inside to outside represent taxonomic levels from phylum to genus. Each small circle at a different taxonomic level represents one taxon at that level, and the diameter of the circle is proportional to the size of the relative abundance. Coloring principle: Species without significant differences are uniformly colored in yellow, different species Biomarker follow the group for coloring, red nodes indicate microbial taxa that play an essential role in the red group, and green nodes indicate microbial taxa that play an essential role in the green group. (**c**) LDA Value Distribution Bar Chart species (The bar chart of the LDA value distribution shows that the LDA score is more than 4), Biomarker with statistically significant differences between groups. Species with significant differences in abundance across groups are shown, and the length of the bar represents the magnitude of the effect of the differing species.

Bacteroidota, Firmicutes, unexplained Bacteria, Proteobacteria, Deferribacteres, Actinobacteria, Ac-tinobacteriota, Cyanobacteria, Verrucomicrobiota, and Fusobacteriota were the phyla (Figure 5a). Bacteroidota and Firmicutes were the most common phyla in the mouse digestive system, accounting for more than 95% of total phyla. The MC group had a lower relative abundance of Firmicutes than the NC group (reduced by 10%). In contrast, Proteobacteria’s relative abundance increased dramatically from 0.1% to 3%. After treatment with PPEAC, the relative abundance of Firmicutes increased by 17%, and Proteobacteria declined dramatically from 3% to 0.2%. It was claimed that PPEAC-H therapy of DSS-induced UC mice could improve gut microorganism structure at the phylum level, bringing it closer to that of the NC group.

LEfSe analysis (Figure 5b,c) revealed that the important biomarkers in the MC group were, in order, g-Bacteroides, g-unidentified-Rhodospirillsles, f-Rikenellaceae, and p-Proteobacteria. In contrast, the significant biomarkers in PPEAC-H were, in order, p-Firmicutes, f-Lachnospiraceae, c-Clostridia, and o-unidentified-Clostridia.

### 2.4. Metabolomic Analysis of PPEAC on Mouse Serum

**Figure 6:**
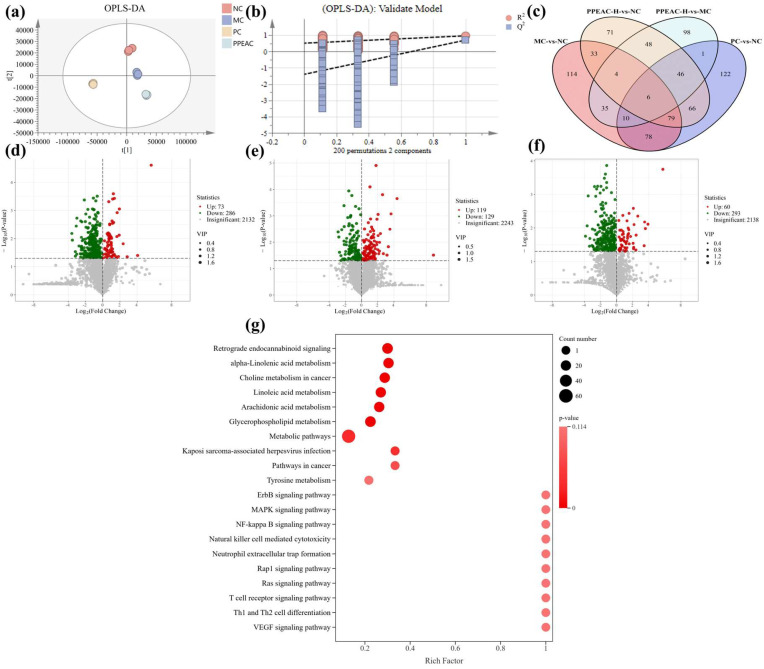
Serum metabolome analysis in mice. (**a**) OPLS-DA. (**b**) 200 cross-validation of OPLS-DA. (**c**) The Venn of differential metabolites between MC and NC, PC and NC, PPEAC-H and NC, and PPEAC-H and MC. (**d**) Volcanic maps of MC and NC, (**e**) Volcanic map of PPEAC-H and NC. (**f**) Volcanic map of PPEAC-H and MC. (**g**) KEGG enrichment bubble plot of differential metabolites in serum.

The intrinsic clustering relationship of the samples in each group can be detected by PCA [9]. The degree of separation between groups was used to perform OPLS-DA analysis using PCA (Figure 6a). OPLS-DA is a supervised discriminant analysis statistical method that helps to better examine the differences among groups [10]. A 200-permutation test was performed on OPLS-DA (Figure 6b), which showed that R2 was close to 1 but not equal to 1, indicating no overfitting, and the model was plausible. The intersection of Q2 with the *y*-axis was negative, indicating a successful model fit. The intervention in the PPEAC-H group effectively distanced the metabolites from the MC group. The screening criteria for serum metabolic differentiators were referred to the study of Liu et al., and the screening criteria were VIP > 1, *p*< 0.05 [11]. Venn diagrams (Figure 6c) were created with the components in each group, which facilitated the visualization of the shared and endemic species of the samples and their numbers, as well as similarities and overlaps in each group. The four groups shared a total of six species: NC and MC, NC and PC, NC and PPEAC-H, and MC and PPEAC-H, with 114, 122, 71, and 98 species endemics to each group. Figure 6d–f show that there were 359 difference metabolites between the NC and MC groups, with 73 up-regulated and 286 down-regulated. The NC and PPEAC-H groups had 353 difference metabolites, of which 60 were up-regulated and 293 were down-regulated, while the MC and PPEAC-H groups had 248 distinct metabolites, 119 of which were up-regulated and 129 were down-regulated.

A total of 248 differential metabolites were identified in the PPEAC-H and MC groups, and evaluating the differences between these two groups may provide a better understanding of the processes by which PPEAC regulates serum metabolism. These 248 differential metabolites were mapped to 134 pathways, and the right panel demonstrated the top 20 pathways (Figure 6g). The significantly enriched pathways (*p* < 0.05) included Kaposi sarcoma-associated herpesvirus infection and metabolic pathways. Pathways with substantial enrichment (*p* < 0.01) included glycerophospholipid metabolism, arachidonic acid metabolism, linoleic acid metabolism, choline metabolism in cancer, alpha-linolenic acid metabolism, and retrograde endocannabinoid signaling.

### 2.5. Effect of PPEAC on Inflammatory Factors in Mouse Serum

**Figure 7:**
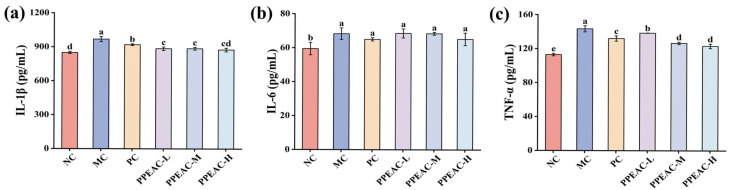
Inflammatory Factors in Mouse Serum. (**a**) IL-1β levels, (**b**) IL-6 levels, (**c**) TNF-α levels. (Significant differences are indicated by different letters in the figure, (*p* < 0.05)).

Serum inflammatory factors, including IL-1β, IL-6, and TNF-α, can cause cellular inflammation and infiltrate intestinal tissues, leading to damage and high levels. This cascade exacerbates UC damage in mice. Immune cells create IL-1β, which encourages the development of additional inflammatory factors [12]. Figure 7a shows that the mice in the MC group had considerably greater levels of IL-1β compared to the NC group (an increase of 119.86 pg/mL, *p* < 0.05). After PC and PPEAC interventions, IL-1β levels were significantly lower than in the MC group, and the PPEAC-H decreased to 875.21 pg/mL, there was no significant difference between the PPEAC-H group and the NC group (848.59 ± 10.35 pg/mL). IL-6, a pro-inflammatory cytokine, prevents the formation of regulatory T cells [13]. Figure 7b shows that the MC group had significantly greater IL-6 levels compared to the NC group (from 59.43 pg/mL to 68.27 pg/mL, *p* < 0.05). IL-6 levels dropped after intervention in the PC and PPEAC groups, but there was no significant difference as compared to the MC group (*p* > 0.05). TNF-α is an inflammatory mediator that disrupts the intestinal barrier, leading to ulcers. Figure 7c demonstrates that the MC group displayed higher levels of TNF-α, whereas both PC and PPEAC considerably reduced its expression (*p* < 0.05). The PPEAC-H group had the most marked effect, surpassing that of the PC group (decreased by 8.95 pg/mL).

### 2.6. Correlation Analysis of Differential Metabolites with Key Strains of Bacteria

**Figure 8:**
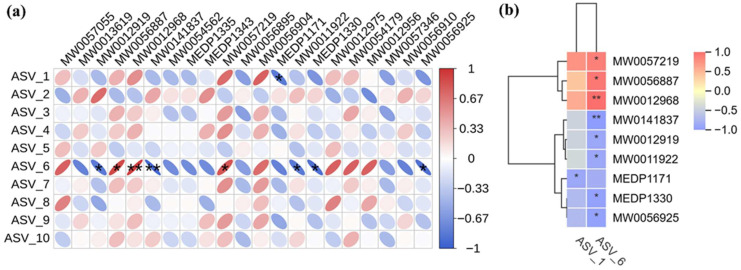
Correlation analysis. (Red means positive correlation, blue means negative correlation, the finer the correlation the greater the absolute value, the darker the color also means the greater the absolute value of the correlation. *: *p* < 0.05, **: *p* < 0.05) (**a**) Corplot plots. (**b**) Clustered heat maps.

Spearman correlation coefficients were employed to further elucidate the therapeutic role of PPEAC, and 21 differential metabolites from serum metabolomics of the MC and PPEAC groups were selected for correlation analysis with the top 10 most abundant species identified in gut flora sequencing. The results of this analysis are presented as corplot plots and clustered heat maps (Figure 8a,b). The results indicated that ASV-1 exhibited a negative correlation (*p* < 0.05) with MEDP1171, while ASV-6 demonstrated significant relationships (*p* < 0.05) with eight substances, comprising three positive and five negative correlations. ASV-6 had a strong positive connection (*p* < 0.01) with MW0012968 and MW0141837, as well as a substantial negative correlation (*p* < 0.01). Tables S1 and S2 describe the 21 differential metabolites from 10 species.

## 3. Discussion

PP is primarily distributed in Southwest China and is recognized as a characteristic resource plant with both edible and medicinal properties [3]. According to folklore, it is believed to be effective in dispelling fire, clearing heat, and possessing anti-inflammatory and detoxifying effects. Research shows that as a functional food, PP can reduce IL-6, IL-1β, and TNF-α levels, leading to anti-inflammatory benefits [6]. However, the anti-inflammatory effects of PP on UC, a common inflammatory condition, have yet to be reported.

In this study, we looked at the therapeutic effects of PPEAC on UC. In a DSS-induced colitis mouse model, we administered three dosages of PPEAC: 125 mg/kg, 250 mg/kg, and 500 mg/kg. To assess the therapeutic efficiency of PPEAC on UC, we evaluated various reliable indicators typically used in colitis research, such as the DAI score, liver ratio, spleen ratio, colon length, and colon oxidative stress. A higher DAI score suggests a more serious UC condition [7]. Our findings revealed a decreasing trend in DAI scores among UC mice treated with PPEAC, suggesting that PPEAC exerts a therapeutic effect on UC, a conclusion that aligns with the research conducted by Ruan [14]. Damage to the intestinal mucosa compromises the integrity of the barrier between the intestines and the bloodstream, resulting in increased intestinal permeability. This allows bacteria and metabolites from the intestines to enter the liver via the bloodstream, causing hepatic damage and an elevated liver ratio [15]. The spleen, as a crucial immune organ, experiences infiltration of inflammatory cells during systemic inflammation, leading to splenomegaly; thus, a higher spleen ratio correlates with increased inflammation [16]. Our results indicated that PPEAC treatment prevented further enlargement of the liver and spleen, suggesting its potential role in alleviating UC. Organ indices serve as critical indicators reflecting the functional integrity of experimental animal organs. By assessing the effects of test substances on these organs, preliminary determinations can be made regarding their toxicity and primary target organs. The results simultaneously confirmed that PPEAC did not induce organ toxicity. Additionally, there is a correlation between colon length and UC severity, with UC mice typically exhibiting shorter colons [17]. This study demonstrated that PPEAC effectively mitigated the reduction in colon length associated with UC. Furthermore, oxidative stress is closely linked to UC; an elevated oxidized level corresponds to increased severity of the condition. This association was further supported by the observed decrease in MDA levels and rise in SOD activity, indicating relief of UC in the current investigation [18]. HE staining showed that PPEAC-treatment markedly reduced inflammatory-cell infiltration, restoring it to a level comparable to that in the NC group Conventional HE staining alone is insufficient to identify goblet cells accurately. Still, the evidence suggests that the treatment may alter the number of them in the intestinal epithelium. In conclusion, PPEAC demonstrated a significant therapeutic effect on UC. Our study demonstrated that in the measured mouse parameters, the 500 mg/kg dose exhibited significantly superior efficacy compared to both 125 mg/kg and 250 mg/kg doses, with 5-ASA showing similar effects. The dose-dependent effects observed across these parameters support the genuine pharmacological action of PPEAC. Furthermore, mice in the PPEAC-H group showed no overt signs of toxicity—such as weight loss, lethargy, or mortality—throughout the 14-day observation period. To further elucidate the therapeutic mechanism of PPEAC, the most effective formulation, PPEAC-H, was selected for serum metabolomics and 16S rRNA sequencing experiments alongside the NC, MC, and PC groups.

Due to the absence of a stable self-equilibrating mechanism in the blood, the concentrations of small-molecule chemicals in the serum exhibit fluctuations within a specific range. These fluctuations reflect the underlying disease processes and external disturbances affecting the organism [19]. Consequently, serum metabolomics plays a crucial role in identifying disease and immune biomarkers. Studies on serum metabolomics in mice have demonstrated alterations in serum composition following PPEAC intervention, with the resulting differential metabolites being analyzed through pathway analysis [20]. Notably, the alpha-linolenic acid metabolism pathway, which has been implicated in the therapy of colitis, was discovered to be greatly enhanced. Disruption of alpha-linolenic acid metabolism leads to reduced anti-inflammatory effects and persistent inflammation. Enhancing alpha-linolenic acid metabolism may help maintain the structural integrity and functionality of tight junctions in intestinal epithelial cells, thereby bolstering intestinal barrier defense. Alpha-linolenic acid (ALA) is a vital polyunsaturated fatty acid that can be metabolized to generate various biologically active substances in vivo. ALA inhibits the NF-kappa B signaling pathway, reducing the generation of pro-inflammatory cytokines TNF-α and IL-6. This lowers intestinal inflammation [21]. Reducing the amounts of inflammatory factors is critical for treating UC [22]. As a result, we examined the inflammatory components in serum and discovered that the PPEAC-H group had much lower levels than the MC group. This finding supports the idea that PPEAC reduces intestinal inflammation by reducing serum inflammatory factors. Modulating the alpha-linolenic acid metabolism pathway, which lowers inflammatory factor synthesis, helps to alleviate UC.

Gut microbiota are key biomarkers and regulators in the pathogenesis of intestinal diseases [23]. The 16S rRNA sequencing data of mice’s intestinal flora revealed that species abundance steadily increased with the administration of PPEAC. Although permutational MANOVA (adonis) based on Bray–Curtis distances did not reach statistical significance (*p* = 0.10), the effect size (R^2^ = 0.312) suggests a biologically relevant separation between MC and PPEAC-H. Consistent with this, Molecular Variance Analysis (AMOVA) detected significant differences among the four groups (*p* = 0.002). The PCoA analysis revealed that the compositional structure of the PPEAC-H group gradually diverged from that of the MC group, implying that PPEAC improved the intestinal flora of mice and that the microbial composition changed when compared to that of MC.

To evaluate the relationship between differential metabolites and the dominant flora in the intestinal flora, we correlated 21 differential metabolites enriched in alpha-linoleic acid metabolism in two groups of sera with the top ten species in relative abundance. Eight of these compounds had high correlations with 2 species. MW0057219, MW0056887, and MW0012968 showed significant positive correlation with species. All three compounds are phosphatidylcholine (PC), which belongs to glycerophospholipids (GP). Phosphatidylcholine (PC) is a significant component of cell membranes with amphipathic molecular properties essential for maintaining the integrity and hydrophobicity of the mucosal barrier [24]. PC is an essential component of the mucus layer in the intestine, forming a hydrophobic barrier to prevent direct contact of harmful substances with intestinal epithelial cells [25]. According to studies, persons with UC have much lower levels of PC in their intestinal mucus than healthy people, which may weaken the barrier and raise the risk of inflammation. Supplementing PC-rich preparations may improve intestinal barrier function in UC patients, lowering inflammation and facilitating illness remission [26].

ASV-1 is classified as Firmicutes. Firmicutes are greatly reduced during the active phase of the disease and recover following remission [27]. The alterations in this species may serve as a biomarker of disease activity. Fecal microbial transplantation (FMT) has been found to play a therapeutic effect in treating UC by restoring the balance of the intestinal microbiota and boosting the quantity of firmicutes [28]. ASV-6 belongs to the Muribaculaceae family of the Bacteroidota, which are widely present in the intestinal microbiota [29]. These bacteria usually attach to the intestinal mucus layer and utilize the glycosides in the mucus as a source of nutrition. In UC patients, the intestinal mucus barrier function is impaired, significantly reducing the abundance of Muribaculaceae [30]. Disruption of the mucus layer reduces the ecological niche of Muribaculaceae but also weakens the intestinal resistance to pathogenic bacteria. Muribaculaceae can metabolize polysaccharides to produce short-chain fatty acids, stimulating mucus release and activating anti-inflammatory signaling pathways, thereby reducing intestinal inflammation [29]. As an important constituent of the intestinal mucus layer, PCs are essential for maintaining the changes in the abundance of Firmicutes and Muribaculaceae. They are closely linked to the function of the intestinal barrier. Decreased PC levels may damage intestinal barrier function, threatening the survival of Firmicutes and Muribaculaceae. Reducing Firmicutes and Muribaculaceae abundance would further weaken the intestinal barrier function, forming a vicious circle. In summary, PPEAC can be therapeutic in UC by increasing PC-like substances, abundant beneficial bacteria such as Firmicutes and Muribaculaceae, and regulating alpha-linolenic acid metabolism.

Although this experiment demonstrated promising therapeutic effects in DSS-induced acute UC mice, its efficacy has not yet been confirmed in chronic or spontaneous UC models. Microbiome and metabolomics analyses remain correlative, lacking functional validation of key strains via transplantation studies. Future work should therefore elucidate the strain–metabolite–target axis. However, before considering any human application, further studies must be conducted, including pharmacokinetic research and large-animal toxicity assessments. Toxicity information on the extract is indispensable for any future clinical translation. Prior to the first human trial, the No Observed Adverse Effect Level (NOAEL) must be determined for the most sensitive animal species based on GLP toxicology studies. This NOAEL should be converted to a human equivalent dose (HED) based on body surface area, incorporating pharmacokinetic data. Subsequently, an appropriate safety factor should be selected based on factors such as the mechanism of toxicity, target organs, and monitorability, to estimate the Maximum Recommended Starting Dose (MRSD). Therefore, in future experiments, PPEAC’s specialized preclinical safety protocol will be prioritized (acute and subchronic toxicity studies).

## 4. Materials and Methods

### 4.1. Materials

PP was collected from wild populations in Lichuan City, Hubei Province, and has been cultivated for several years at the experimental farm of Hubei Xiongzhan Agricultural Co., Ltd. (Lichuan, China). Its identity was verified morphologically by Prof. Yi Yongmei (College of Forestry and Horticulture, Hubei Minzu University, China). The chemicals and reagents utilized in the investigation were analytically pure.

### 4.2. Preparation of PPEAC

Freshly harvested PP leaves were cleaned and dried at 55 °C in a blast drying oven before being pulverized and sieved through a 60-mesh sieve to produce PP leaf powder. A specific weight of PP powder was measured, and an ultrasonic extraction was performed at 200 W with a material-to-liquid ratio of 1:40 g/mL, using 77% ethanol as the solvent. The extraction was carried out for 15 min at room temperature. After the resultant alcoholic extracts were concentrated, the extract was dissolved by adding distilled water at a material-to-liquid ratio of 1:10 g/mL. The mixture was then extracted with an equal volume of ethyl acetate and freeze-dried to obtain the PPEAC solid.

### 4.3. Metabolomic Analysis of PPEAC

Building on the modified methodology from Ye et al. [31], freeze-dried PPEAC was ground into a powder using a grinder operating at 30 Hz for 1.5 min. A 50 mg powdered sample was weighed, and mixed with 1200 μL of a precooled 70% aqueous methanol solution at −20 °C. Vortexing was executed for 30 s every 30 min, resulting in six vortexing sessions. The supernatant was gently aspirated after centrifugation at 12,000 rpm for 3 min. The filtered sample was then placed in an injection vial for UPLC-MS/MS analysis after passing through a microporous membrane with a pore size of 0.22 μm. This study included three biological replicates. The UPLC-MS/MS conditions were shown in Tables S3 and S4.

### 4.4. Experimental Design

#### 4.4.1. Modeling Methods for UC Mice

The experimental animals were C57BL/6 mice (male, 7 weeks old, body weight 20 ± 2 g, Liaoning Changsheng Biotechnology Co., Shenyang, China). After one week of acclimatization and feeding in a sterile environment with a temperature of 22–25 °C, a relative humidity of 60–70%, and a 12 h light/dark cycle, the animals were randomly assigned into six groups: Normal Control (NC), Model Control (MC), and Positive Control (PC) groups received 125 mg/kg 5-ASA, PPEAC-L (125 mg/kg), PPEAC-M (250 mg/kg), and PPEAC-H (500 mg/kg), with 10 mice per group. All groups, except the NC group, were administered a 3% DSS solution ad libitum for 7 days [32]. The PPEAC group was gavaged with the corresponding concentration of PPEAC. Before administering medication via the gastric tube, we use ultrasound equipment to dissolve the powdered PPEAC in saline solution. This work was approved by the ethical committee of Hubei Minzu University (No. 2023084).

#### 4.4.2. Measurement of DAI in Mice

The DAI is calculated by adding the scores for weight loss, fecal characterization, and fecal occult blood [33]. The indexes were recorded after the start of modeling. Table 1 presented the criteria used for DAI scoring.

#### 4.4.3. Determination of Liver and Spleen Ratio and Colon Length in Mice

The mice were weighed once before execution and recorded. After the mice were put to death, their livers, spleens, and colons were removed, weighed, and recorded sequentially, The following formulas were then used to determine the liver index and spleen index: liver ratio = liver weight/body weight; spleen ratio = spleen weight/body weight [34].

#### 4.4.4. Measurement of Oxidative Stress Levels in Mice Colons

The kit was purchased from Shanghai Jining Shiye Co., Ltd. (Shanghai, China) The specific measurement method is as follows:

The method for determining SOD enzyme activity involves weighing approximately 0.1 g of tissue, adding 1 mL of extraction solution, and homogenizing on an ice bath. Centrifuge at 8000× *g* and 4 °C for 10 min, then collect the supernatant. Following the kit instructions, sequentially add 50 μL sample extract (replace with 50 μL distilled water in the control group), 50 μL Reagent III, 800 μL working solution, and 100 μL Reagent IV. Mix thoroughly. After incubating at room temperature for 30 min, measure the absorbance of each tube at 560 nm.

The method for determining MDA content is as follows: Weigh approximately 0.1 g of tissue, add 1 mL of extraction solution, and homogenize on an ice bath. Centrifuge at 8000× *g* and 4 °C for 10 min. Remove the supernatant. Pipette 0.6 mL of Reagent 1 into a 1.5 mL centrifuge tube, then add 0.2 mL of the supernatant and mix thoroughly. Place in a 95 °C water bath for 30 min, then cool in an ice bath. Centrifuge at 10,000× *g* and 4 °C for 10 min. Transfer the supernatant to a 1 mL cuvette and measure the absorbance at 532 nm and 600 nm.

#### 4.4.5. Hematoxylin & Eosin (H&E) Stain

The colon was preserved with a 4% neutral paraformaldehyde solution, embedded in paraffin, and sectioned at 5 μm. The sections were stained with hematoxylin and eosin, and the histological changes in the colon were examined under a microscope.

### 4.5. Intestinal Flora Studies in Mice

The intestinal flora was sequenced using the previously established research method with modifications [35]. In summary, the collection of mouse feces by tail-carrying method prior to euthanasia was placed in sterilized freezing tubes and frozen at −80 °C for subsequent 16S rRNA sequencing analysis. A small library of fragments was created according to the characteristics of the amplified 16S region and underwent paired-end sequencing on the Illumina Nova Seq platform (San Diego, CA, USA). The resulting reads were filtered through splicing, and representative sequences were generated by applying clustering or noise reduction methods for species annotation and abundance analysis. The data were stored in the NCBI database (PRJNA1179378).

### 4.6. Serum Metabolomics Studies

In light of a prior investigation with specific alterations, the process involved removing samples from the −80 °C storage unit and allowing them to thaw on ice until they were completely free of ice crystals. Following the thawing, the samples were vortexed and mixed for 10 s, after which 50 μL of each sample was transferred into appropriately labeled centrifuge tubes. Subsequently, 300 μL of a 20% acetone-triple-methanol internal standard extract was incorporated, and vortexing was conducted for 3 min. The mixture was then centrifuged at 12,000 rpm for 10 min at a temperature of 4 °C. Post-centrifugation, 200 μL of the supernatant was carefully pi-petted into another appropriately labeled centrifuge tube and allowed to stand for 30 min at −20 °C. The subsequent step involved another centrifugation for 10 min at 4 °C and a final centrifugation at 12,000 rpm for 10 min. Eventually, 200 μL of the supernatant was transferred into a tube designated for the corresponding UPLC-MS/MS analysis. The UPLC-MS/MS conditions were shown in Tables S5 and S6.

### 4.7. Measurement of Serum Inflammatory Factor Levels in Mice

Following the successful establishment of the DSS-induced mouse model, blood was taken from the mice’s orbital venous plexus and allowed to stand for 24 h. The serum was then isolated and kept at −80 °C. Serum levels of inflammatory cytokines IL-1β, IL-6, and TNF-α were measured using enzyme-linked immunosorbent assay (ELISA).

### 4.8. Data Processing

The data was analyzed using SPSS 26.0 statistical software, and the significance of differences was determined using one-way ANOVA. Results were given as “mean ± standard error” for significant differences (*p* < 0.05) and extremely significant differences (*p* < 0.01). In addition, the graphs were created with Origin Pro 2024, Simca 14.0 software, and Mavis Cloud Platform (https://cloud.metware.cn/).

## 5. Conclusions

The results demonstrated that PPEAC exhibited favorable therapeutic effects on UC. PPEAC could reduce Disease Activity Index (DAI) scores, and the liver and spleen ratios, attenuate colonic shortening and pathological damage, and reduce oxidative damage. Furthermore, we found that PPEAC also can reduce the inflammatory factors in serum. Manifesting as a dose dependent response across all measured indicators.

Serum metabolomics research has found that it was observed to regulate the alpha-linolenic acid metabolism pathway, thereby contributing to the therapeutic management of UC. The results of the joint gut microbiota analysis indicate: PPEAC was discovered to improve the intestinal environment of mice by raising the quantity of PC-like compounds and boosting the abundance of beneficial bacteria, such as Firmicutes and Muribaculaceae. This study offers new insights for treating UC, and establishes a solid foundation for the use of PP in the treatment of UC.

## Figures and Tables

**Table 1:** DAI Scoring Criteria.

Index	Weight Loss/%	Fecal Trait	Fecal Blood
0	0%	Normal	Negative
1	1–5%	Loose and tangible	Weak positive
2	5–10%	Loose	Positive
3	10–15%	Very loose; damp	Strong positive
4	>15%	Loose stools	Bloody stool

## Data Availability

The data presented in this study are available on request from the corresponding author due to privacy restrictions.

## Supplementary Materials

The following supporting information can be downloaded at: https://www.mdpi.com/article/10.3390/molecules30183809/s1, Figure S1: Total Ion Chromatogram of PPEAC in Positive Ion Mode. Figure S2: Total Ion Chromatogram of PPEAC in Negative Ion Mode. Table S1: 21 differential metabolites enriched into alpha-linoleic acid metabolism. Table S2: Top 10 species in abundance in gut flora sequencing. Table S3: HPLC Conditions. (A = Water containing 0.1% Formic acid acetonitrile, B = Acetonitrile containing 0.1% Formic acid). Table S4: MS Conditions. Table S5: HPLC Conditions. (A = Water containing 0.1% Formic acid acetonitrile, B = Acetonitrile containing 0.1% Formic acid). Table S6: MS Conditions.
