# Supplementary material for: Premna puberula P. Ethyl Acetate Extract Treats Ulcerative Colitis by Regulating the Intestinal Flora and Improving Serum Metabolism
Source: Molecules. 2025 Sep 19;30(18):3809. doi: 10.3390/molecules30183809 (PMC12472188; doi:10.3390/molecules30183809)
Supplement: Supplementary file 1 [file molecules-30-03809-s001.zip › molecules-3828626-supplementary.pdf]

Supplementary Materials

Premna puberula P. Ethyl Acetate Extract Treats Ulcerative Colitis by Regulating the Intestinal Flora and Improving Serum Metabolism

Zhichao Wang [1], Yanmei Zhang [2], Yun Huang [2], Qiang Xiao [3], Yuchang Zhu [1,3,]* and Dazhai Zhou [2,3,]*

[1] College of Biological and Food Engineering, Hubei Minzu University, Enshi 445000, China; 202330402@hbmzu.edu.cn

[2] College of Forestry and Horticulture, Hubei Minzu University, Enshi 445000, China;
18845569341@163.com (Y.Z.); 202430445@hbmzu.edu.cn (Y.H.)

[3] Hubei Key Laboratory of Biological Resources Protection and Utilization, Hubei Minzu University,
Enshi 445000, China; 1992022@hbmzu.edu.cn

* Correspondence: 2006025@hbmzu.edu.cn (Y.Z.); 2001033@hbmzu.edu.cn (D.Z.)

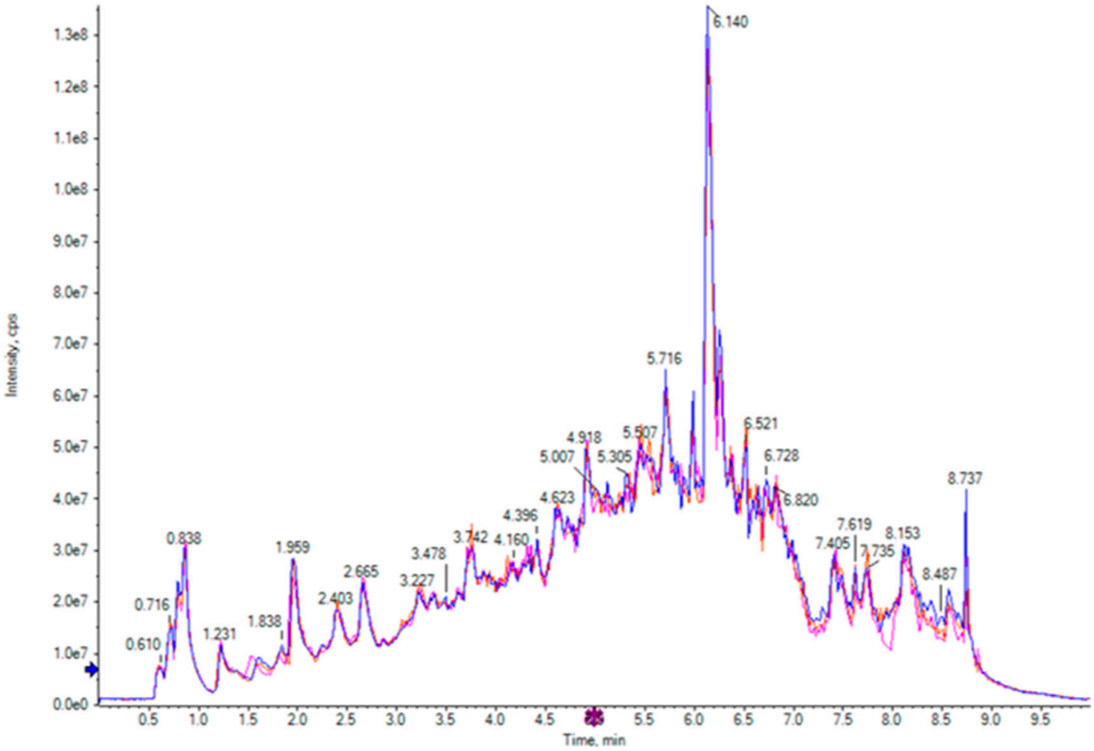

Figure S1. Total Ion Chromatogram of PPEAC in Positive Ion Mode.

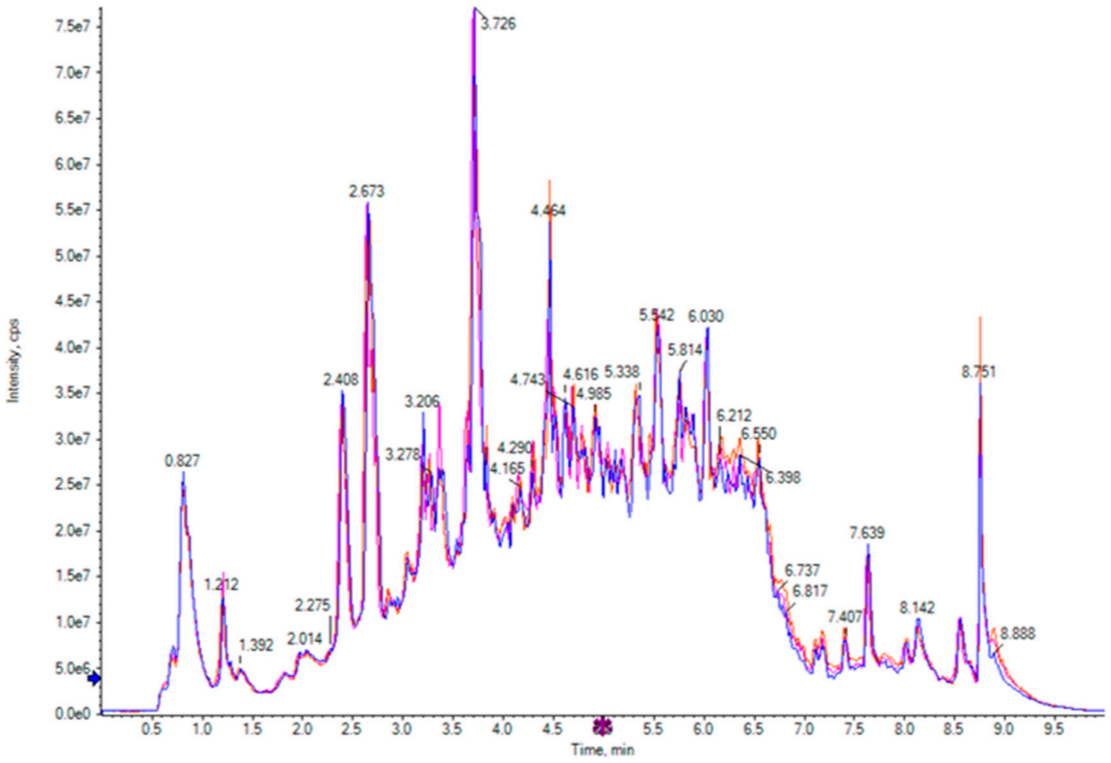

Figure S2. Total Ion Chromatogram of PPEAC in Negative Ion Mode.

Table S1. 21 differential metabolites enriched into alpha-linoleic acid metabolism.

Index	Compounds	Class	Formula	VIP	P-value	Fold_Change	Type
MW0057055	1,2-Dilinoleoyl-SN-glycero-3-phosphocholine	GP	$C_{44}H_{80}NO_8P$	1.44	0.01	4.13	up
MW0013619	1-O-Octadecyl-2-O-methyl-sn-glycero-3-phosphocholine	GP	$C_{27}H_{58}NO_6P$	1.74	0.00	0.33	down
MW0012919	LPE(18:0/0:0)	GP	$C_{23}H_{48}NO_7P$	1.59	0.02	0.39	down
MW0056887	1-Palmitoyl-2-oleoyl-sn-glycero-3-phosphocholine	GP	$C_{42}H_{82}NO_8P$	1.72	0.03	8.83	up
MW0012968	1-hexadecanoyl-2-(9Z,12Z-octadecadienoyl)-sn-glycero-3-phosphocholine	GP	$C_{42}H_{80}NO_8P$	1.72	0.01	9.91	up
MW0141837	1-Stearoyl-2-Arachidonoyl PC-d8	GP	$C_{46}H_{76}D_8NO_8P$	1.69	0.00	0.40	down
MW0054562	1-(1Z-hexadecenyl)-sn-glycero-3-phosphocholine	GP	$C_{24}H_{50}NO_6P$	1.47	0.05	0.56	down
MEDP1335	LPC(20:5/0:0)	GP	$C_{28}H_{48}NO_7P$	1.51	0.05	0.65	down
MEDP1343	LPC(0:0/18:3)	GP	$C_{26}H_{48}NO_7P$	1.40	0.05	0.61	down
MW0057219	1-(11Z-eicosenoyl)-2-(9Z-octadecenoyl)-glycero-3-phosphocholine	GP	$C_{46}H_{88}NO_8P$	1.46	0.03	1.75	up
MW0056895	PC(16:0/20:3(5Z,8Z,11Z))	GP	$C_{44}H_{82}NO_8P$	1.68	0.03	0.17	down
MW0056904	1-hexadecanoyl-2-(4Z,7Z,10Z,13Z,16Z-docosapentaenoyl)-sn-glycero-3-phosphocholine	GP	$C_{46}H_{82}NO_8P$	1.53	0.00	20.84	up
MEDP1171	Butenoyl-PAF	GP	$C_{28}H_{56}NO_7P$	1.71	0.00	0.40	down
MW0011922	1,2-Dipentadecanoyl-sn-glycero-3-phosphocholine	GP	$C_{38}H_{76}NO_8P$	1.60	0.04	0.33	down

MEDP1330	LPC(0:0/20:2)	GP	$C_{28}H_{54}NO_7P$	1.68	0.00	0.48	down
MW0012975	1-Hexadecanoyl-2-octadecanoyl-sn-glycero-3-phosphocholine	GP	$C_{42}H_{84}NO_8P$	1.45	0.00	11.31	up
MW0054179	Jasmonic acid	Hormones	$C_{12}H_{18}O_3$	1.54	0.01	3.66	up
MW0012956	PC(16:0/20:4(5Z,8Z,11Z,14Z))	GP	$C_{44}H_{80}NO_8P$	1.76	0.03	469.55	up
MW0057346	PC(20:4(5Z,8Z,11Z,14Z)/15:0)	GP	$C_{43}H_{78}NO_8P$	1.46	0.04	0.68	down
MW0056910	1-Palmitoyl-2-(1-enyl-palmitoyl)-sn-glycero-3-phosphocholine	GP	$C_{40}H_{80}NO_7P$	1.44	0.02	0.34	down
MW0056925	PC(16:1(9Z)/18:4(6Z,9Z,12Z,15Z))	GP	$C_{42}H_{74}NO_8P$	1.72	0.00	0.69	down

Table S2. Top 10 species in abundance in gut flora sequencing.

Index	Phylum	Class	Order	Family	Genus	Species
ASV_1	Firmicutes	Clostridia	unidentified_Clostridia	Lachnospiraceae	-	-
ASV_2	Firmicutes	Bacilli	Erysipelotrichales	Erysipelotrichaceae	Faecalibaculum	Faecalibaculum_rodentium
ASV_3	Bacteroidota	Bacteroidia	Bacteroidales	Muribaculaceae	-	-
ASV_4	Bacteroidota	Bacteroidia	Bacteroidales	Muribaculaceae	-	-
ASV_5	Bacteroidota	Bacteroidia	Bacteroidales	Prevotellaceae	unidentified_Prevotellaceae	Alloprevotella_sp_feline_oral_taxon_309
ASV_6	Bacteroidota	Bacteroidia	Bacteroidales	Muribaculaceae	-	-
ASV_7	Firmicutes	Clostridia	unidentified_Clostridia	Lachnospiraceae	Anaerostipes	-
ASV_8	Bacteroidota	Bacteroidia	Bacteroidales	Prevotellaceae	Paraprevotella	-
ASV_9	Firmicutes	Clostridia	unidentified_Clostridia	Lachnospiraceae	-	-
ASV_10	Bacteroidota	Bacteroidia	Bacteroidales	Muribaculaceae	-	-

Table S3. HPLC Conditions. (A=Water containing 0.1% Formic acid acetonitrile, B=Acetonitrile containing 0.1% Formic acid)

Time (min)	A (%)	B (%)
0.0	95	5
5.0	35	65
6.0	1	99
7.5	1	99
7.6	95	5
10.0	95	5

Table S4. MS Conditions.

Mass spectrometry conditions	ESI+	ESI-
Duration (min)	10	10
IonSpray Voltage (V)	5000	-4000
Temperature (°C)	550	550
Ion Source Gas1 (psi)	50	50
Ion Source Gas2 (psi)	60	60
Curtain Gas (psi)	35	35
Declustering Potential (V)	60	-60
MS1 Collision Energy(V)	10	-10
MS2 Collision Energy(V)	30	-30
Collision Energy Spread(v)	15	15
MS1 TOF Masses(Da)	50-1250	50-1250
MS2 TOF Masses(Da)	25-1250	25-1250

Table S5. HPLC Conditions. (A=Water containing 0.1% Formic acid acetonitrile, B=Acetonitrile containing 0.1% Formic acid)

Time (min)	A (%)	B (%)
0.0	95	5
2.0	80	20
5.0	40	60
6.0	1	99
7.5	1	99
7.6	95	5
10.0	95	5

Table S6. MS Conditions.

Mass spectrometry conditions	ESI+	ESI-
Duration (min)	10	10
IonSpray Voltage (V)	5000	-4000
Temperature (°C)	550	550
Ion Source Gas1 (psi)	50	50
Ion Source Gas2 (psi)	60	60
Curtain Gas (psi)	35	35
Declustering Potential (V)	60	-60
MS1 Collision Energy(V)	10	-10
MS2 Collision Energy(V)	30	-30
Collision Energy Spread(v)	15	15
MS1 TOF Masses(Da)	50-1000	50-1000
MS2 TOF Masses(Da)	25-1000	25-1000
MS1 Accumulation time(s)	0.2s	0.2s
MS2 Accumulation time(s)	0.04s	0.04s
Candidate ions	18	18
